# Developmental mechanisms underlying circuit wiring: Novel insights and challenges ahead

**DOI:** 10.1016/j.conb.2020.12.013

**Published:** 2021-01-06

**Authors:** Heike Blockus, Franck Polleux

**Affiliations:** 1 Department of Neuroscience, Columbia University, New York, NY 10027, USA; 2 Zuckerman Mind Brain Behavior Institute, Columbia University, New York, NY 10027, USA; 3 Kavli Institute for Brain Science, Columbia University, New York, NY 10027, USA

## Abstract

Synaptic connectivity within neural circuits is characterized by high degrees of cellular and subcellular specificity. This precision arises from the combined action of several classes of molecular cues, transmembrane receptors, secreted cues and extracellular matrix components, coordinating transitions between axon guidance, dendrite patterning, axon branching and synapse specificity. We focus this review on recent insights into some of the molecular and cellular mechanisms controlling these transitions and present the results of large-scale efforts and technological developments aimed at mapping neural connectivity at single cell resolution in the mouse cortex as a mammalian model organism. Finally, we outline some of the technical and conceptual challenges lying ahead as the field is starting to explore one of the most challenging problems in neuroscience: the molecular and cellular logic underlying the emergence of the connectome characterizing specific circuits within the central nervous system of mammals.

## Mapping the complexity of the wiring diagram characterizing functional circuits

Brain development is an extraordinarily complex process for any organism to achieve properly. It can be broken down into subsequent steps starting from proliferation, specification and differentiation of neuronal and glial progenitor cells, then cell migration, axon guidance and branching, dendritic patterning and synapse formation. Both activity-independent [1,2] and activity-dependent mechanisms [3,4] interplay for the refinement of synaptic connectivity during neuronal maturation. During and to some extent following critical periods, synapses and neurons display various forms of functional and structural plasticity, allowing the organism to learn and adapt to its environment. However, orchestrating such strikingly different biological processes during brain development is endowed to a relatively limited set of genes. This is especially remarkable in light of the complexity of the wiring diagram characterizing functional circuits. The central nervous systems (CNS) of invertebrates and vertebrates including mammals is complex at multiple levels of organization. First, the diversity of neuronal cell types defined in terms of gene expression, dendritic morphology (postsynaptic sampling field), axon projections (presynaptic sampling field), synaptic connectivity and electrophysiological properties is staggering. Over the past decade or so, the emergence of techniques such as single cell RNA sequencing (scRNAseq) has revealed the existence of high degrees of neuronal subtypes diversity, at least defined transcriptionally [5]. For example, when comparing mouse and human cerebral cortex, several studies have converged on the existence of ~20 excitatory long-projecting neuronal subtypes and ~40 inhibitory neuronal subtypes [6•]. Whether or not each of these transcriptionally defined neuronal subtypes corresponds to individual or multiple subclasses of neurons defined in terms of connectivity and electrophysiological properties [7] is a matter of intense investigation (see for example [8,9]).

**Figure 1:**
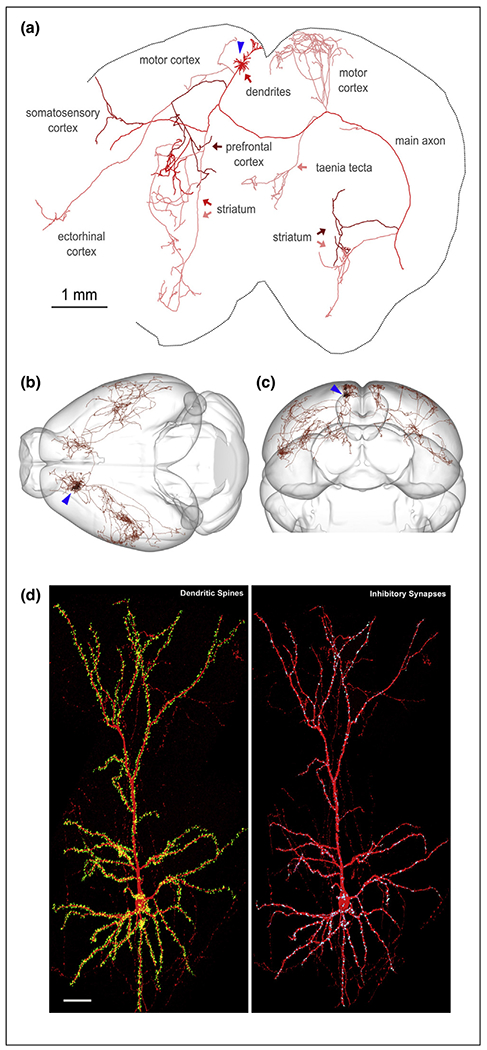
Striking complexity of neuronal connectivity. **(a–c)** Two-photon serial tomography coupled with computational approaches allows the complete tracing of the axon projections of a single layer 5 pyramidal neurons in the mouse motor cortex (cell body position indicated by blue arrow in a–c). Panel A shows a partial, compressed, 2D representation of the axon of this PN which projects to 8 different structures distributed throughout the entire mouse brain (a). Panels (b) and (c) show the complete 3D structure of the axon projection of the same neuron from a dorsal (b) and posterior view (c). **(d)** Complete 3D reconstruction and annotation of all excitatory synapses (dendritic spines, yellow in left panel) and inhibitory (I) synapses labeled with Gephyrin-EGFP (blue in right panel) in optically isolated layer 2/3 PNs of the primary somatosensory cortex. This particular neuron receives 8115 E synapses and 1045 I synapses. Scale bar in D: 50 microns. Panels (a–c) are reproduced with permission from Ref. [16] and the Mouselight Project. Panel (d) reproduced with permission from Ref. [24•].

Recent large scale efforts to use serial electron microscopy to map all neuronal connections (connectomics) characterizing circuits of the central nervous system have been restricted to rather compact brains of invertebrate model organisms such as *Drosophila* melanogaster (see recent reviews [10–12]). In larger vertebrate brains and in particular the central nervous system (CNS) of mammals, previous studies have started to map the remarkable degree of complexity characterizing neuronal connectivity within circuits. For example, single cell anterograde and monosynaptic viral tracing demonstrated the extreme degree of divergence and lack of stereotypy characterizing the axonal projections of single mitral cells from the mouse olfactory bulb to the pyriform cortex [13–15]. More recent large-scale efforts to map the pattern of axonal projections and connectivity of individual neurons in the mouse brain have confirmed that this remarkable degree of complexity in the projection pattern of individual neurons is the rule rather than the exception. For example, reconstructions of axonal projections of single neurons have shown that a significant proportion of long-range projecting pyramidal neurons (PNs) such as layer 5 PNs of the mouse cortex project to up to 8–12 individual cortical and subcortical targets simultaneously [16,17•] (Figure 1a–c). The MouseLight and the Allen Mouse Brain Connectivity Atlas represent large scale efforts to map the pattern of connectivity characterizing individual neurons or groups of neurons in various regions of the mouse brain. The immense challenge for the field is to relate the axonal projection patterns of individual neuronal subtypes to their electrophysiological properties and transcriptional identity.

Similarly, in the mouse visual cortex, anatomical evidence suggested the existence of at least 9 retinotopically organized visual areas outside V1 (area 17) [18]. Each of these areas display unique patterns of visual responses and selectivity [19,20]. Recent improvements in single cell axon tracing technologies, such as whole brain serial 2-photon tomography [21] and MapSeq [22], have allowed to test if individual cortico-cortical (CC) PNs in V1 projects to these 9 secondary visual areas according to (1) a ‘dedicated output’ model where individual neurons projects primarily to one area outside V1, or (2) a ‘random broadcasting’ model in which CCPNs subtypes project to a random combination of 1–9 areas simultaneously through axon branching or (3) a ‘broadcasting motifs’ model where axonal projections of individual CC PNs subtypes display biased projections to a limited subset of areas. The answer seems to be the latter in the mouse visual cortex where a significant fraction of individual CC PNs project to a biased and limited number of secondary visual areas and therefore suggests the existence of a limited number of broadcasting motifs [23••].

Conversely, Iascone *et al.* recently mapped the postsynaptic distribution of over 90 000 E and I synapses received by twelve L2/3 PNs and uncovered structured organization of E and I synapses across dendritic domains as well as within individual dendritic segments in these neurons [24•]. Despite significant, domain-specific, variations in the absolute density of E and I synapses, their ratio is strikingly balanced locally across dendritic segments. As shown in Figure 1D, this example layer 2/3 PNs receives 8115 E synapses and 1045 I synapses originating from ~20 cortical and extracortical regions as revealed using sparse Rabies monosynaptic tracing in the same neuronal subtype [25].

## Molecular and cellular logic underlying circuit wiring in the mammalian CNS

The immense challenge facing the field is to answer the major question that emerges from these recent investigations: what are the molecular and cellular mechanisms underlying the establishment of these patterns of connectivity among neurons defining functional circuits? When examining the pattern of axon projection of a neuron such as the layer 5 PN in the mouse motor cortex shown in Figure 1a–c, the answer is far from obvious based on our current knowledge.

**Figure 2:**
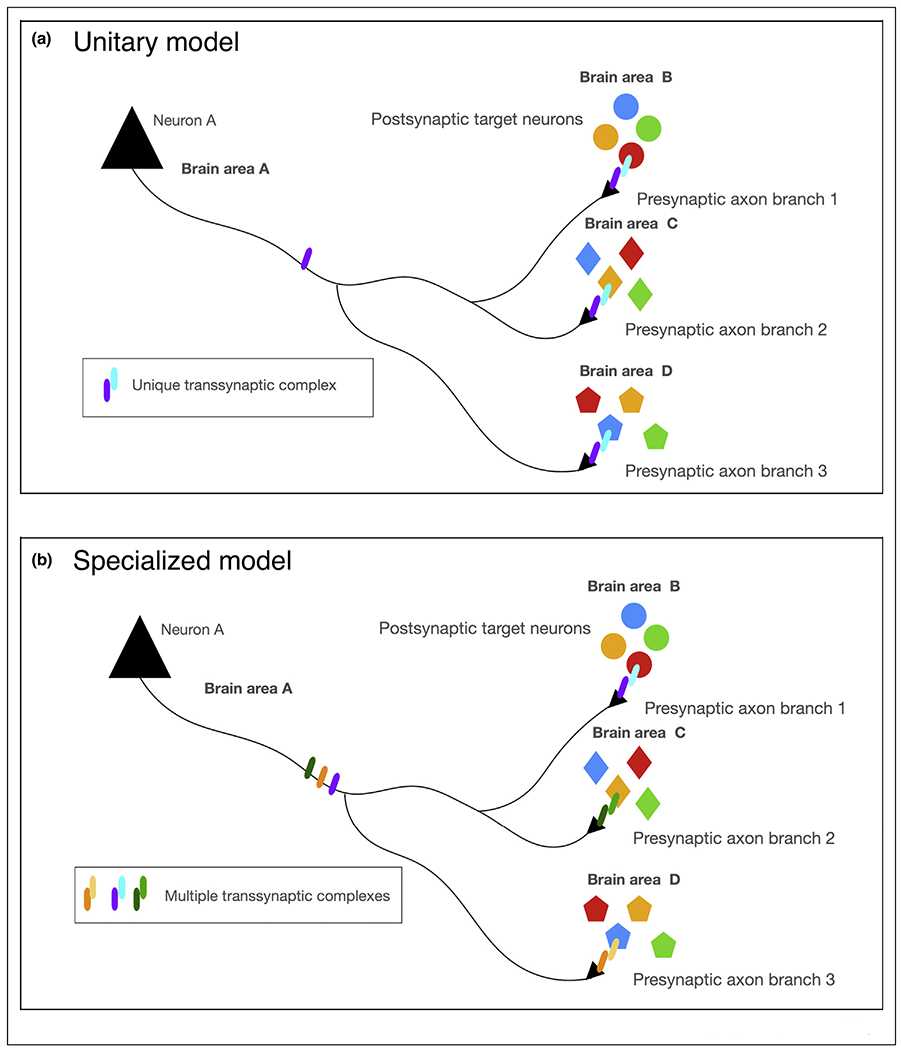
Potential molecular models underlying the establishment of synaptic specificity along a single axon projecting to multiple brain structures within a distributed circuit. Neuron A projects to three different brain structures where it contacts three different neuronal subtypes. What molecular mechanism could underlie the establishment of synaptic specificity in such a distributed circuit? **(a)** A *unitary* molecular model of axonal connectivity where the same unique transynaptic protein complex underlies synaptic specificity in each target region with diverse postsynaptic target neurons. **(b)** A *distributed* molecular model where distinct transynaptic protein complexes mediate the establishment of synaptic specificity in a branch-specific way which would require distinct transynaptic molecular effectors to be targeted and/or locally translated in a branch-specific way. See text for details.

Controlling the projection pattern of an axon connecting with ~10 distinct structures scattered throughout the brain is not a ‘simple’ axon guidance problem: how is each axon collateral of a given neuron responds to presumably distinct axon guidance cues following the formation of each interstitial branch? How does the branching pattern characterize each of these axon collaterals that are regulated independently? Once reaching each distinct target, how do individual axon branches establish synapses with completely different subtypes of postsynaptic neurons? One could imagine two extreme models: (1) a ‘unitary’ molecular model whereby each axon branch forms synapses with this distributed network of postsynaptic neurons determined by expression of the same set of synaptogenic cues, regardless of the postsynaptic target. Therein, a single combination of trans-synaptic protein complexes would exist at these synapses, matching presynaptic axon branches to postsynaptic dendrites or dendritic subdomains (Figure 2a) or (2) a ‘specialized’ molecular model, whereby each axon branch would be able to form synaptic contacts with their distributed postsynaptic target neurons based on various different sets of synaptogenic cues expressed in a cell-type (postsynaptically) and branch-specific manner (presynaptically) (Figure 2b). In the latter model, each axon branch would have to control the expression and/or membrane presentation of the presynaptic proteins required to form the trans-synaptic complexes with appropriate postsynaptic neurons with each targets. Local protein translation which is a prominent feature of growing dendrites and axons [26] could help increase specificity of expression of synaptogenic cues, or their downstream signaling components, in a branch-specific way (see for example [27•]).

Our current knowledge of the mechanisms regulating axon guidance, terminal axon branching and synaptic specificity only offer very partial answers to these challenging problems and we will review some of them below.

## Molecular mechanisms underlying synaptic specificity

Once axons have reached each of their postsynaptic target field, the need to form synapses with precise degree of both cell-type and sub cellular specificity. Significant progress has been made recently in the identification of trans-synaptic protein complexes that orchestrate the precise wiring of these hippocampal circuits. Several synaptic adhesion molecules show strikingly confined expression patterns across laminae in CA1 PNs (review in Ref. [28]). Among these, some of the best characterized family of trans-synaptic proteins playing key roles in establishing synaptic specificity are Neurexins [29] and Leucine-Rich Repeat-domain containing TransMembrane proteins (LRRTMs) [30,31]. These synaptogenic proteins constitute molecular recognition motifs that have the unique ability to trigger assembly of the presynaptic release machinery (for example by Neurexins) and postsynaptic molecular scaffolding of glutamate receptors at excitatory synapses or GABA receptors at inhibitory synapses [32].

These trans-synaptic interactomes can generate a large number of combinatorial interactions, which together with alternative splicing [33] and local protein synthesis [26] might underlie complex patterns of synaptic connectivity. Here, we highlight recent findings focusing on the development of the mouse hippocampus, a circuit characterized by exquisite laminar segregation of its inputs lends itself perfectly to the mechanistic study of cellular and subcellular synaptic specificity.

## Emergence of synaptic specificity in developing hippocampal circuits

**Figure 3:**
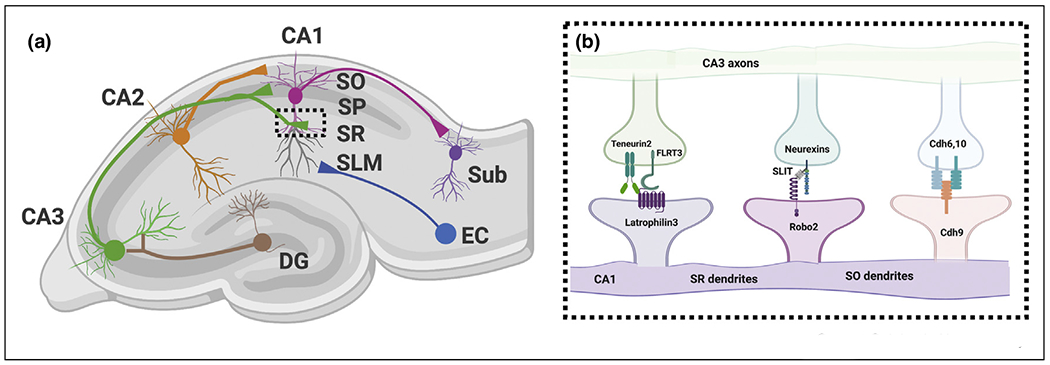
Molecular mechanisms underlying synaptic specificity of CA3 > CA1 connectivity. Hippocampal circuit diagram detailing connections between the cornus ammonis (CA) regions and the entorhinal cortex (EC), Subiculum (Sub) and Dentate gyrus (DG). Inset: Overview of synaptic adhesion molecules implicated in the development of synaptic specificity within hippocampal CA3 → CA1 projections. Abbreviations: stratum oriens (SO), stratum radiatum (SR), stratum lacunosum moleculare (SLM), granule cells (GCs).

The hippocampus is a cortical structure in the temporal lobe, most prominently known for its role in learning and memory. Its exquisite laminar organization makes the hippocampus an excellent model to investigate how input-specific compartmentalization of axons onto dendritic arbors of its principal neurons arises during development (Figure 3a). Pyramidal neurons (PNs) in the CA1 region of the hippocampus are well-characterized for their unique activity patterns that encode location-specific information. CA1 PNs that display heightened activity in specific locations are termed ‘place cells’ [34]. Place cell formation relies on the spatio-temporal integration of axonal inputs from the entorhinal cortex (EC) onto distal apical tuft dendrites of CA1 PNs in the SLM (*stratum lacunosum moleculare*) region and axonal input from CA3 and CA2 PNs onto proximal dendritic compartments, apical and basal, in SR (*stratum radiatum*) and SO (*stratum oriens*), respectively (Figure 3a).

Several synaptic adhesion molecules show strikingly confined expression patterns across laminae in CA1 PNs (review in Ref. [28]). For example, Latrophilin3 (Lphn3) is an adhesion-GPCR previously characterized as a synaptogenic protein (refs) and its expression is restricted to the basal dendrites (SO) and apical oblique (SR) of CA1 PNs but absent from the apical tufts of these neurons (SLM). Conversely, Lphn2 is enriched in the apical tuft (SLM) of CA1 PNs [35,36••]. Using combination of conditional knockout approaches, slice electrophysiology and rabies monosynaptic tracing, Sando *et al.* [36••] recently provided evidence that Lphn3 is required for establishment of synaptic specificity in the CA3 → CA1 connectivity. Cell-autonomous, postsynaptic deletion of FLRT2 or Lphn3 expression in CA1 PNs lead to a loss of about ~50% of synapses in CA1:SO/SR (Figure 3a) [36••,^37^]. Postsynaptic function of Lphn3 relies on coincident binding of presynaptic FLRT3 and Ten2, illustrating that formation of multimeric complexes increases the realm of unique synaptic specificity recognition.

A recent study identified another trans-synaptic complex formed by postsynaptic Robo2, its soluble ligand Slit and presynaptic Neurexins in the formation of CA3 → CA1 connectivity [38••]. Robo2 has been characterized extensively for its role in axon guidance in the developing brain of many model organisms [39]. Robo2 protein is expressed in CA1 PNs in a strikingly restricted manner (present in SO and SR but absent from SLM) and is enriched postsynaptically. Using biochemistry, conditional knockout approaches, slice electrophysiology and *in vitro* synaptogenic assays, Blockus *et al.* demonstrate that Robo2 promotes the formation of E (but not I) synapses in a Slit-binding and Neurexin-binding dependent way. Interestingly, sparse, cell-autonomous conditional deletion of Robo2 leads to ~40% loss of dendritic spines in SO and SR but not in SLM arguing that postsynaptic Robo2 is required for formation of almost half of CA3 → CA1 inputs. Using *in vivo* 2-photon Ca^2+^ imaging in awake behaving mice, Blockus *et al.* demonstrate that CA1-specific, conditional deletion of Robo2 leads to significant alterations in place cell properties (reduction in fraction of spatially tuned cells, increase in fraction of ‘silent’ cells) compared to control CA1 PNs in the same animals. These results provide a unique link between the molecular mechanisms underlying synaptic specificity and the emergence of neuronal subtype-specific response properties and circuit function. These results also point to the pleiotropy of protein function in the developing CNS, where proteins such as Robo2 can regulate axon guidance mostly through chemorepulsion and promote excitatory synapse formation through formation of a unique tripartite trans-synaptic complex with Slit and Neurexins. Previous axon guidance cues have been involved in regulating various aspects of synaptic development but further investigations will be required to understand the molecular mechanisms underlying their context-dependent switch in function during this key transition between axon guidance and synapse formation (see review in this issue [40]).

The results from Sando *et al.* [36••] and those of Blockus *et al.* [38••], suggest that deletion of two completely distinct trans-synaptic molecular complexes (Lphn3/Ten/FLRT and Robo2/Slit/Nrxn) both lead to ~40–50% loss of CA3 → CA1 synapses. This poses the question whether Lphn3 and Robo2localize to different subsets of spines and/or receive input from different (previously unknown) subpopulations of axons from CA3 PNs. To address this, monosynaptic rabies tracing from neurons deficient in a given synaptic adhesion complex would enable identification of potential molecularly defined subcircuits within the hippocampus even within one of the best-studied connection in the mammalian CNS, the CA3 → CA1 circuit. Future experiments will be needed to determine how the proteins forming these trans-synaptic complexes are so strikingly restricted at the subcellular level for example in subdomains of the dendritic arbor of CA1 PNs.

## Conclusion

A recent study illustrates the remarkable degree of complexity characterizing molecular composition of transynaptic protein complexes [41••]. The authors managed to purify a single type of synapse, one of the largest in the mammalian central nervous system: the mossy fiber originating from DG granule cells forming synapses with a specialized postsynaptic protrusion called the thorny excrescences in the proximal portion of the dendrite of CA3 PNs. Remarkably, using proteomic approaches, this study identified and validated a panel of 77 cell-surface proteins (CSPs) including adhesion proteins, receptors, secreted glycoproteins, receptor protein tyrosine phosphatases and tyrosine kinases [41••]. Future investigations will need to identify the role of the other ~70 cell surface proteins present at this single synapse and determine if this degree molecular complexity controls synapse-specific functions such as presynaptic release properties and pre- or postsynaptic expression of plasticity. Another possibility to explain this extreme molecular diversity at one synapse is that many of these proteins form multimeric molecular complexes increasing the specificity of protein–protein interactions underlying synaptic specificity.
